# Autophagy in periodontitis patients and gingival fibroblasts: unraveling the link between chronic diseases and inflammation

**DOI:** 10.1186/1741-7015-10-122

**Published:** 2012-10-17

**Authors:** Pedro Bullon, Mario David Cordero, José Luis Quiles, Maria del Carmen Ramirez-Tortosa, Adrian Gonzalez-Alonso, Simona Alfonsi, Rocio García-Marín, Manuel de Miguel, Maurizio Battino

**Affiliations:** 1 Department of Periodontology, Dental School, University of Seville, c/Avicena s/n, Sevilla, 41009, Spain; 2 Departamento de Citología e Histología Normal y Patológica, Facultad de Medicina, Universidad de Sevilla, Avenida Sanchez Pizjuan s/n, Sevilla, 41009, Spain; 3 Department of Physiology, Institute of Nutrition and Food Technology 'José Mataix', Biomedical Research Center, University of Granada, Avenida del Conocimiento s/n, Armilla Granada, 18100, Spain; 4 Department of Biochemistry and Molecular Biology II, Institute of Nutrition and Food Technology 'José Mataix', Biomedical Research Center, University of Granada, Avenida del Conocimiento s/n, Armilla Granada, 18100, Spain; 5 Dipartimento di Scienze Biomediche e Sanità Pubblica - Sezione di Anatomia Patologica Università, Università Politecnica delle Marche, Via Tronto, 10, Ancona, 60100, Italia; 6 Dipartimento di Scienze Cliniche Specialistiche ed Odontostomatologiche - Sezione Biochimica, Università Politecnica delle Marche, Via Ranieri, 65, Ancona, 60100, Italia

## Abstract

**Background:**

Periodontitis, the most prevalent chronic inflammatory disease, has been related to cardiovascular diseases. Autophagy provides a mechanism for the turnover of cellular organelles and proteins through a lysosome-dependent degradation pathway. The aim of this research was to study the role of autophagy in peripheral blood mononuclear cells from patients with periodontitis and gingival fibroblasts treated with a lipopolysaccharide of *Porphyromonas gingivalis*. Autophagy-dependent mechanisms have been proposed in the pathogenesis of inflammatory disorders and in other diseases related to periodontitis, such as cardiovascular disease and diabetes. Thus it is important to study the role of autophagy in the pathophysiology of periodontitis.

**Methods:**

Peripheral blood mononuclear cells from patients with periodontitis (n = 38) and without periodontitis (n = 20) were used to study autophagy. To investigate the mechanism of autophagy, we evaluated the influence of a lipopolysaccharide from *P. gingivalis *in human gingival fibroblasts, and autophagy was monitored morphologically and biochemically. Autophagosomes were observed by immunofluorescence and electron microscopy.

**Results:**

We found increased levels of autophagy gene expression and high levels of mitochondrial reactive oxygen species production in peripheral blood mononuclear cells from patients with periodontitis compared with controls. A significantly positive correlation between both was observed. In human gingival fibroblasts treated with lipopolysaccharide from *P. gingivalis*, there was an increase of protein and transcript of autophagy-related protein 12 (ATG12) and microtubule-associated protein 1 light chain 3 alpha LC3. A reduction of mitochondrial reactive oxygen species induced a decrease in autophagy whereas inhibition of autophagy in infected cells increased apoptosis, showing the protective role of autophagy.

**Conclusion:**

Results from the present study suggest that autophagy is an important and shared mechanism in other conditions related to inflammation or alterations of the immune system, such as periodontitis.

## Background

An appreciation of the rising global burden of chronic, noncommunicable diseases has grown in the last years. Cardiovascular disease (CVD) is one of the leading causes of death and disability worldwide, accounting for 16.7 million (29.2%) of total global deaths [1]. Abundant evidence has demonstrated that reducing modifiable CVD risk factors (smoking, lipid fractions, blood pressure, diabetes) through drug, dietary and other interventions can prevent or delay CVD events. Although implementation of clinical preventive guidance is improving over time, there is still a large proportion of coronary patients who do not reach the lifestyle, risk factor and therapeutic targets for CVD prevention [2]. Therefore, some other approach should be implemented. The new approach could come from the study of the pathologic mechanisms involved in CVD.

Periodontitis is generally a chronic disorder characterized by the breakdown of tooth-supporting tissues, producing a loss of dentition. It is the most prevalent chronic inflammatory human disease, affects 30% to 40% of the population over 35 years of age, and is considered a major problem in the global burden of oral diseases [3]. The cause is an ecological imbalance between the microbial biofilm on teeth and an impaired host inflammatory response. The disease involves the breakdown of the gingival connective tissue, namely gingival fibroblast dysfunction. It has been related to CVD; for instance, periodontitis is significantly associated with the risk of developing cerebrovascular incidents and, in particular, nonhemorrhagic stroke [4]. In a recent Editor's Consensus Report between *The American Journal of Cardiology *and *Journal of Periodontology*, this interrelationship was reviewed and future research was requested to find the best management to reduce CVD risk in periodontitis [5].

Inflammation needs the proper functioning of cells. The degradation of damaged and excess organelles as well as the elimination of invading pathogens is essential to maintain cell homeostasis. Autophagy is the principal catabolic pathway allowing the cell to survive the stress of these and other intrinsic and extrinsic insults [6]. The autophagy machinery interfaces with most cellular stress-response pathways, including those involved in controlling immune responses and inflammation [7]. Impaired autophagy is correlated with various severe pathologies, including cardiovascular and autoimmune diseases [8]. More specifically, constitutive autophagy in the heart under baseline conditions is a homeostatic mechanism for maintaining cardiomyocyte size and global cardiac structure and function [9]. The molecular mechanism underlying autophagy has been extensively researched in the past decade, and the genes participating in this process, usually named autophagy-related genes (ATGs) [10], were found to be conserved in yeast and humans [11,12]. Oxidative stress has been shown to induce autophagy under starvation and ischemia/reperfusion conditions [13,14]. Within most cells, the mitochondrion is the main source of reactive species which are by-products of cell energy production. All conditions able to alter mitochondria efficiency can enhance the production of reactive oxygen species (ROS), having a direct and critical effect on oxidative stress [15].

Periodontitis as an example of a chronic inflammatory disease could share autophaghic alterations. On the one hand, in the oral environment, the inflammatory response is often evocated by specific bacteria, like *Porphyromonas gingivalis*. On the other hand, oxidative stress is one of the main factors explaining the pathophysiological mechanism of inflammatory conditions that occur in atherosclerosis and periodontitis. Several studies have demonstrated an increase of products from oxidative damage in plasma and serum in patients with periodontitis compared with healthy individuals [16,17]. Moreover, there is evidence both for a decreased antioxidant capacity in patients with periodontitis, evaluated by different assays [18-20].

Evidence has been found indicating a regulatory role for ROS of mitochondrial origin as signaling molecules in autophagy, leading, under different circumstances, to either survival or cell death [21,22]. Recently, our group reported high levels of mitochondrial-derived ROS, accompanied by mitochondrial dysfunction in peripheral blood mononuclear cells (PBMCs) from patients with periodontitis [23]. Furthermore, *P. gingivalis *lipopolysaccharide (LPS) was found to be responsible for high mitochondrial ROS and coenzyme Q10 (CoQ_10_) levels and for mitochondrial dysfunction because it affected the amount of respiratory chain complex I and III. Therefore, LPS-mediated mitochondrial dysfunction could be the reason for oxidative stress onset in patients with periodontitis.

The purpose of the present study was two-fold. First, to investigate if periodontitis, as a chronic inflammatory disease, modifies the autophagy capacity of PBMCs. Second, to elucidate, in an *in vitro *model with gingival fibroblasts, in what way bacterial periodontal infection with *P. gingivalis *LPS alters autophagy mechanisms, and if this process should be considered a protective rather than a pathological mechanism.

## Methods

### Ethics statement

The study was approved by the Ethics Review Board of the University of Seville. All the studies involving human participants were conducted in full compliance with government policies and the Declaration of Helsinki. All participants completed an informed consent.

### Patients

Patients attending Seville University Dental School over a period of 10 months were invited to participate in the study. A total of 65 consecutive patients, all over 35 years old, agreed to participate and signed the written consent form. Protocol and consent forms had been previously approved by the Committee of Ethics and Research of Seville University (16 December 2006). All patients met the following inclusion criteria: they had more than 20 teeth, they had not taken antibiotics or anti-inflammatory drugs in the previous six months, they were not affected by immunodeficiency, and were generally healthy and had undergone no previous periodontal treatment. Exclusion criteria were acute infectious diseases during the previous three weeks; past or present neurological, psychiatric, metabolic, autoimmune or allergy-related problems, undesired habits (for example, smoking, alcohol); medical conditions that required glucocorticoid treatment or use of analgesics, statin or antidepressant drugs; past or current substance abuse or dependence; and pregnancy or current breastfeeding. A blood sample was taken from each patient as they were recruited.

A baseline periodontal examination was performed, and a single examiner collected full medical and dental histories. A single trained dental examiner recorded periodontal data. Periodontal probing depth (PD) from the gingival margin (GM) to the most apical penetration of the probe and the position of the GM relative to the cement enamel junction were measured at six sites per tooth. Clinical attachment level (CAL) was calculated by adding GM to PD. PD and CAL were recorded to the nearest highest millimeter by means of the North Carolina periodontal probe (Hu-Friedy, Chicago, IL, USA), 15 mm in length and 0.35 mm in diameter. The proportion of sites positive for plaque and for bleeding on probing were obtained for each patient. According to the criteria established by Machtei *et al*. [24], the clinical entity of periodontitis is based on the presence of CAL ≥6 mm in two or more teeth and one or more sites with PD ≥5 mm. Fifty-eight potential participants met the inclusion criteria and were enrolled in the study, and seven patients were excluded: three smoked cigarettes, three were using antidepressant treatment, and one was using statins. Patients were divided into two groups: one with periodontitis (n = 38) and the other without periodontitis (n = 20), who were healthy controls. Healthy controls had no sign or symptom of periodontitis, and had a healthy status and were free of any medication for at least three weeks before the study began.

### Reagents and chmicals

Mitosox, LysoTracker and Hoechst 3342 were purchased from Invitrogen/Molecular Probes (Eugene, OR, USA); anti-hATG12 from Biosensis (South Australia, Australia); anti-MAP LC3 (N-20) from Santa Cruz Biotechnology (Santa Cruz, CA, USA); a cocktail of protease inhibitors from Boehringer Mannheim (Indianapolis, IN, USA); and Immun Star HRP substrate kit from Bio-Rad Laboratories Inc. (Hercules, CA, USA). Monoclonal anti-actin antibodies, butylated hydroxyanisole (BHA), N-acetylcysteine (NAC), 3-methyl adenine (3-MeA), trypsin-EDTA solution and all other chemicals were purchased from Sigma-Aldrich (St. Louis, MO, USA).

### Blood mononuclear cell and fibroblast cultures

Heparinized and coagulated blood samples were collected from each patient, centrifuged at 3800 ×g for 5 min, and plasma and serum stored separately at -80°C. PBMCs were purified by isopycnic centrifugation using Histopaque-1119 and Histopaque-1077 (Sigma Chemical Co., St. Louis, MO, USA).

Human gingival fibroblasts (HGF) isolated from a healthy 25-year-old man were cultured in D-MEM media (4500 mg/L glucose, L-glutamine, pyruvate), (Gibco, Invitrogen, Eugene, OR, USA) supplemented with 10% FBS (Gibco) and antibiotics (Sigma Chemical Company). Cells were incubated at 37°C in a 5% CO_2 _atmosphere. HGF were cultured with 10 μg/mL LPS of *P. gingivalis *(Nucliber S.A., Spain). When required, CoQ_10_, alpha tocopherol, BHA and NAC were added to the plates at a final concentration of 30 μM, 10 μM, 40 μM and 10 mM, respectively.

### Mitochondrial reactive oxygen species production

Mitochondrial ROS generation in PBMCs and fibroblasts were assessed by MitoSOX Red, a red mitochondrial superoxide indicator. MitoSOX Red is a novel fluorogenic dye recently developed and validated for highly selective detection of superoxide in the mitochondria of living cells [25]. MitoSOX Red reagent is live-cell permeant and is rapidly and selectively targeted to the mitochondria. Once in the mitochondria, MitoSOX Red reagent is oxidized by superoxide and exhibits red fluorescence.

#### Flow cytometry

Approximately 1 × 10^6 ^cells were incubated with 1 μM MitoSOX Red for 30 min at 37°C, washed twice with PBS, resuspended in 500 μL of PBS and analyzed by flow cytometry in an Epics XL cytometer (Beckman Coulter, Brea, California, USA; excitation at 510 nm and fluorescence detection at 580 nm).

#### Fluorescence microscopy

Cells grown on microscope slides in six-well plates for 24 h were incubated with MitoSOX Red for 30 min at 37°C, washed twice in PBS, fixed with 4% paraformaldehyde in PBS for 0.5 h to 1 h, and washed twice with PBS. Cells were then incubated for 10 min at 37°C with anti-LC3 antibody (Santa Cruz Biotechnology). Slides were analyzed by immunofluorescence microscopy (MitoSOX Red: excitation wavelength = 555/28; emission wavelength = 617/73).

### Western blotting for autophagy protein

Whole cell lysate from HGF was prepared by gently shaking the cells with a buffer containing 0.9% NaCl, 20 mM Tris-HCl, pH 7.6, 0.1% triton X-100, 1 mM phenylmethylsulfonylfluoride and 0.01% leupeptin. Electrophoresis was carried out in a 10% to 15% acrylamide SDS-PAGE. Proteins were transferred to Immobilon membranes (Amersham Pharmacia, Piscataway, NJ, USA) and, after blocking overnight at 4°C, incubated with the respective antibody solution, diluted at 1:1,000. Membranes were then probed with their respective secondary antibody (1:2,500). Immunolabeled proteins were detected by using a chemiluminescence method (Immun Star HRP substrate kit, Bio-Rad Laboratories Inc.). Protein was determined by the Bradford method [26].

### β-galactosidase test

Autophagy induces an increment of degradative enzymes mediated by lysosomal activity; therefore, to evaluate lysosomal β-galactosidase protein, cultured HGF were washed in PBS (pH 7.4), fixed with 3.7% formaldehyde and incubated overnight at 37°C in freshly prepared staining buffer (1 mg/mL Xgal (5-bromo-4-chloro-3-indolyl β-D-galactosidase), 5 mM K_3_Fe[CN]_6_, 5 mM K_4_Fe[CN]_6 _and 2 mM MgCl_2 _in PBS, pH 6.0, or in citrate-buffered saline, pH 4.5). At the end of incubation, cells were washed with PBS, examined, and photographed using a Leica CTR 5000 microscope. β-galactosidase staining was quantified using Image J software (Bethesda, MD, USA).

### LysoTracker Red assay for acidic lysosomes

LysoTracker (100 nM) was added to cultured HGF as a fluorescent acidotropic probe for labeling and tracking acidic organelles. After 30 min, cells were harvested, incubated with fresh medium, washed, centrifuged (500 ×g), and resuspended in DMEM medium. The red fluorescence of LysoTracker was quantified by flow cytometry in an Epics XL cytometer (Beckman Coulter; excitation wavelength: 577 nm, emission wavelength: 590 nm) [27].

### Real-time quantitative PCR of autophagy genes

Total cellular RNA was purified from the cultured cells using the Trisure method (Bioline, London, UK), according to the manufacturer's instructions. RNA concentration was determined using spectrophotometry. To avoid genomic DNA contamination, one microgram of total RNA from each sample was incubated in gDNA wipe out buffer (Quantitect Reverse Transcription Kit, Qiagen, Hilden, Germany) at 42°C for 5 min. RNA samples were subsequently retrotranscribed to cDNA using QuantiTect Reverse Transcription Kit (Qiagen). Quantitative RT-PCRs were performed in a miniopticon unit (Bio-Rad) making use of the SensiMix One-Step qRT-PCR Kit (London, UK). The thermal cycling conditions used were: denaturation at 95°C for 20 s, alignment at 54°C for 20 s and elongation at 72°C for 20 s, for 40 cycles. Human ATG12 primers 5'-ATTGCTGCTGGAGGGGAAGG-3' (forward primer) and 5'-GGTTCGTGTTCGCTCTACTGC-3' (reverse primer) amplify a sequence of 198 nucleotides. Human MAP-LC3 primers 5'-GCCTTCTTCCTGCTGGTGAAC-3' (forward primer) and 5'-AGCCGTCCTCGTCTTTCTCC-3' (reverse primer) amplify a sequence of 91 nucleotides.

### Electron microscopy

HGF were fixed for 15 min in the culture plates with 1.5% glutaraldehyde in culture medium, then for 30 min in 1.5% glutaraldehyde-0.1 M Na cacodylate/HCl, pH 7.4. They were then washed three times in 0.1 M Na cacodylate/HCl, pH 7.4 for 10 min and post-fixed with 1% OsO_4_-H_2_O, pH 7.4 for 30 min. After dehydration process during 5 min in each increasing concentrations of ethanol (50%, 70%, 90% and three times 100%), impregnation steps and inclusion were performed in Epon and finally polymerized at 60°C for 48 h. An ultramicrotome was used to obtain 60 nm to 80 nm sections (Leica Ultracut S; Leitz Microsystems, Wetzlar, Germany) and contrasted with uranyl acetate and lead citrate. Observations were performed on a Zeiss LEO 906 E transmission electron microscope (Zeiss, Oberkochen, Germany).

### Proliferation rate

Two hundred thousand HGF were cultured with LPS (10 μg/mL) in the absence or presence of 3-MeA (20 mM) for 24 h. After discharging the supernatant with dead cells, cell counting was performed from three high power fields using an inverted microscope and a 40× objective.

### Analysis of apoptosis

Apoptosis in HGF treated with LPS was assessed by observing nuclei fragmentation by Hoechst staining (0.05 μg/ml), as previously described [28].

### Statistical analysis

All results are expressed as means ± SD unless stated otherwise. An unpaired Student's t test was used to evaluate the significance of differences between groups, accepting *P *< 0.05 as the level of significance. Statistical analyses included Pearson's correlations between mitochondrial ROS in PBMCs from patients, and MAP-LC3 expression levels; *P *< 0.05 were considered significant. Data were analyzed using the SPSS/PC statistical software package (SPSS for Windows, 19, 2010, SPSS Inc. Chicago, IL, USA).

## Results

### Clinical data

Of the 58 patients who met the inclusion criteria and agreed to participate, 38 were diagnosed with periodontitis. Table 1 summarizes the results of the periodontal examination with significant differences in all the parameters studied (*P *< 0.001 for GM, PD, CAL, dental plaque and gingival bleeding determinations) while no significant differences were found for age and body mass index between the considered groups.

**Table 1:** Periodontal data in patients with and without periodontitis.

	Periodontis (n = 38)	Non-periodontis (n = 20)
Age (years)	40 ± 9	41.1 ± 5
Body mass index (kg/m^2^)	25.7 ± 1.7	24.6 ± 1.2
*Periodontal data*		
GM (mm)	0.8 ± 0.07^a^	0.17 ± 0.02
PD (mm)	3.3 ± 0.5^a^	1.5 ± 0.3
CAL (mm)	4.1 ± 0.3^a^	2.2 ± 0.3
Dental plaque (%)	48.1 ± 3.7^a^	21.1 ± 2.2
Gingival bleeding (%)	61.9 ± 4.1^a^	38.3 ± 4.1

Data represent the mean ± SD. CAL: clinical attachment level; GM: position of the gingival margin; PD: periodontal probing depth. ^a ^Significantly different periodontitis versus non-periodontitis: P < 0.001.

### Reactive oxygen species-dependent autophagy in patients with periodontitis

**Figure 1:**
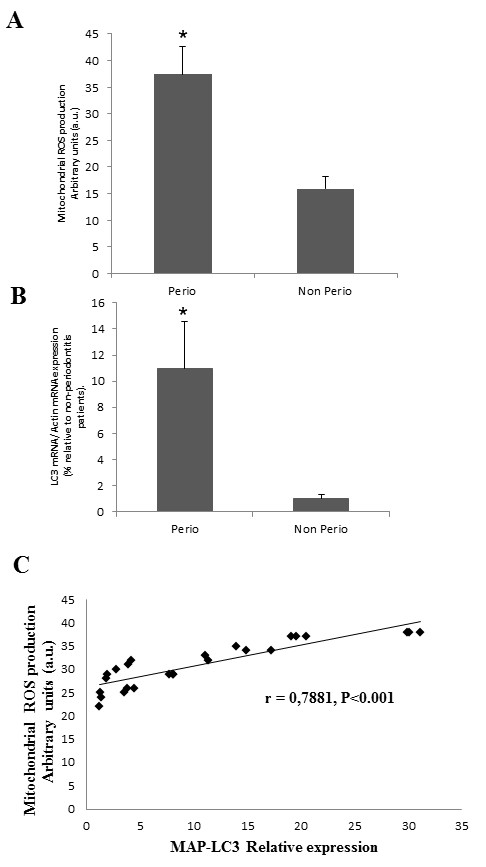
**Reactive oxygen species production and autophagy in periodontitis patients**. **(A) **ROS production was analyzed in PBMCs from patients with and without periodontitis by flow cytometry as described in Methods. *P < 0.001 periodontitis versus no periodontitis. **(B)**. Expression of LC3 transcripts in PBMCs from patients with and without periodontitis assessed by real-time PCR as described in Methods.*P <0.001 periodontitis versus no periodontitis. **(C) **Correlation between ROS levels and MAP-LC3 mRNA levels in PBMCs from patients with periodontitis. Data represent the mean ± SD of three separate experiments. non perio: participants without periodontitis; PBMC: peripheral blood mononuclear cells; perio: patients with periodontitis; ROS: reactive oxygen species; SD: standard deviation.

Quantification of ROS production by flow cytometry analysis showed high levels of mitochondrial ROS production in PBMCs from patients with periodontitis compared with patients without periodontitis (Figure 1A). To observe the presence of autophagy in patients with periodontitis, we analyzed the expression of the gene *LC3*, which encodes a protein involved in autophagic processes. PBMCs from patients with periodontitis showed a significantly increased expression of MAP-LC3 gene compared with those from controls (Figure 1B). To further examine the role of ROS generation in autophagy present in periodontitis, we statistically analyzed the correlation between both parameters. A significant positive correlation (r = 0.7881, *P *< 0.001) was observed showing the role of ROS in autophagy in periodontitis (Figure 1C).

### Lipopolysaccharide induces autophagy in human gingival fibroblasts

**Figure 2:**
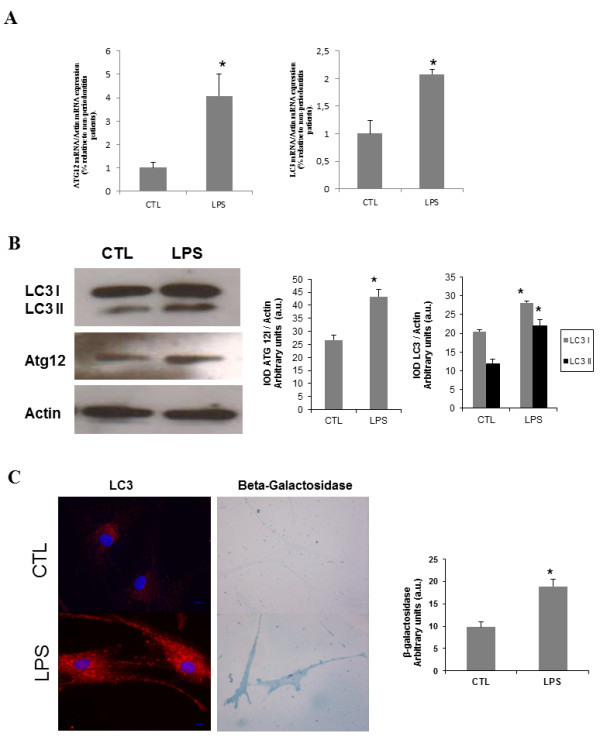
**Autophagy in human gingival fibroblasts treated with lipopolysaccharide (10 μg/mL)**. **(A) **mRNA levels of ATG12, and LC3 in control and LPS-treated fibroblasts. Statistical significance: *control versus LPS-treated HGF (*P *< 0.01) **(B) **Protein expression of Atg12 and LC3. Protein levels were determined by densitometric analysis of three different western blots and normalized to GADPH signal.*P < 0.01, between control and LPS-treated fibroblasts. **(C) **Representative images of autophagic markers (LC3, β-galactosidase) in control and LPS-treated fibroblasts that were visualized by immunofluorescence and light microscopy respectively, as described in Methods. Bar = 25 μm. Data represent the mean ± SD of three separate experiments. CTL: control; IOD: integrated optical intensity; LPS: lipopolysaccharide.

**Figure 3:**
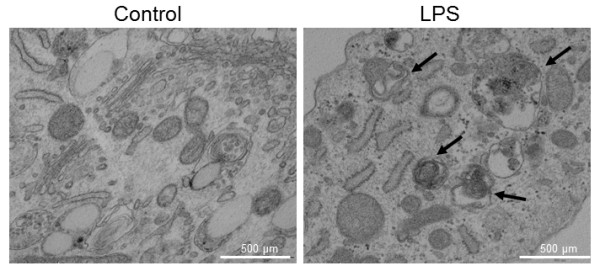
**Ultrastructure of lipopolysaccharide-treated human gingival fibroblasts (10 μg/mL)**. Control fibroblasts show mitochondria with typical ultrastructure. Laminar bodies and autophagosome with mitochondria were present in LPS-treated fibroblasts (black arrow); Bar = 500 nm. LPS: lipopolysaccharide.

Activation of LPS-induced autophagy was observed when analyzing the expression of the genes *ATG12 *and *LC3 *by analyzing mRNA levels and protein expression. HGF treated with LPS showed a significantly increased mRNA level for both studied genes, compared with control HGF (Figure 2A). The protein level of ATG12 was also increased in LPS fibroblasts (Figure 2B). We investigated the relative protein abundance of LC3-I and LC3-II, the ratio of which represents the conversion of LC3-I to L3-II and is considered a marker of autophagic activity. We found a significant increase in LC3-II conversion in LPS HGF, indicating enhanced autophagosome formation (Figure 2B). Autophagy was further verified under microscopic analysis by increased intensity, due to β-galactosidase and LC3 (Figure 2C). To confirm the presence of mitochondrial degradation or mitophagy in LPS fibroblasts, we performed electron microscopy on control and LPS-treated fibroblasts. Figure 3 clearly shows the presence of autophagosomes and laminar bodies in LPS-treated HGF, indicating extensive autophagy. In early autophagosomes it is possible to see mitochondria that are being degraded.

### Lipopolysaccharide-induced autophagy in human gingival fibroblasts depends on reactive oxygen species

**Figure 4:**
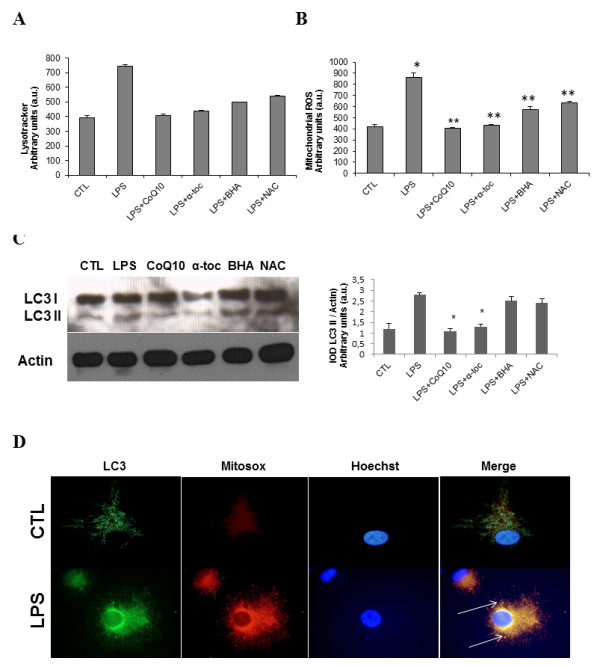
**Action of antioxidants on human gingival fibroblast-related autophagy and mitochondrial co-localization of lysosomal markers of autophagy**. **(A) **Quantification of acidic vacuoles in control and LPS-treated fibroblasts by Lysotracker staining and flow cytometry analysis after antioxidant treatment.*P < 0.01 between control and LPS-treated fibroblasts.**P < 0.01 between LPS-treated fibroblasts and LPS + antioxidants. **(B) **Quantification of mitochondrial ROS in control and LPS-treated fibroblasts by MitoSOX staining and flow cytometry analysis after antioxidant treatment.*P < 0.01 between control and LPS-treated fibroblasts.**P < 0.01 between LPS-treated fibroblasts and LPS + antioxidants. **(C) **Protein expression of LC3 in HGF treated with LPS 10 μg/mL and antioxidants performed by western blotting as described in Methods. **P < 0.001 between LPS-treated fibroblasts and LPS + antioxidants. **(D) **Mitochondrial-induced ROS degraded by autophagy. Mitochondrial ROS production was localized by Mitosox Red staining. Cells were then harvested, fixed and immunostained with LC3 (autophagosome marker) and examined in a fluorescence microscope as described in Methods. Data represent the mean ± SD of three separate experiments. α-toc: α-tocopherol; BHA: butylated hydroxyanisole; CTL: control; CoQ_10_: coenzyme Q_10_; HGF: human gingival fibroblasts; LPS: lipopolysaccharide; NAC: N-acetylcysteine.

To further examine the role of ROS generation in LPS-induced autophagy, we cultured HGF in the presence of LPS and, alternatively, four antioxidants: CoQ10, α-tocopherol, BHA and NAC. We quantified levels of acidic vacuoles by using Lysotracker staining, mitochondrial ROS production by MitoSOX and flow cytometry analysis. We observed that acidic vacuoles and ROS were significantly increased in LPS-treated HGF with respect to controls. Interestingly, all antioxidants significantly attenuated autophagy (Figure 4A) and ROS (Figure 4B). We also investigated the conversion of LC3-I to LC3-II after antioxidant treatment. Figure 4C shows a significant decrease in LC3-II conversion in LPS HGF, indicating a reduction in autophagosome formation. Immunofluorescence studies, staining with antibodies against LC3, also indicated that autophagosome accumulation co-localized with a mitochondrial superoxide marker, such as MitoSOX Red (Figure 4D).

### Lipopolysaccharide-induced autophagy could play a protective role

**Figure 5:**
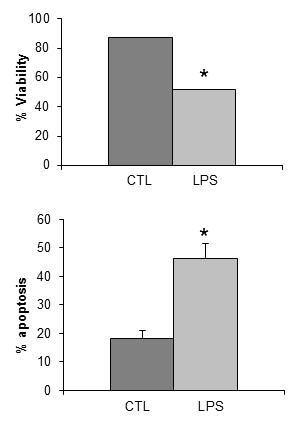
**Indices of cell viability and apoptosis in control and lipopolysaccharide-treated fibroblasts after autophagy arrest by 3-methyl adenine (20 mM)**. Results are expressed as mean ± SD of three independent experiments. *P < 0.01 between control and LPS-treated fibroblasts. CTL: control: LPS: lipopolysaccharide.

To elucidate whether autophagy in LPS-treated HGF was a protective or a pathological mechanism, we examined the effect of blocking autophagy by using 3-MeA (20 mM), a well-characterized inhibitor of the early stages of autophagy. We examined LPS-treated and control HGF for viability and apoptosis. Figure 5 clearly shows that inhibiting autophagy in LPS-treated HGF provides, as a concomitant result, a significant reduction of cell viability and an increase of the apoptosis rate.

## Discussion

The main finding of the present study is that autophagy might be an important mechanism involved in chronic inflammatory diseases like periodontitis. Here, we have demonstrated, for the first time in patients with periodontitis, an enhancement of the autophagy phenomenon mediated by mitochondrial ROS in PBMCs. Also, our *in vitro *gingival fibroblast model showed how the periodontal etiological agent *P. gingivalis *LPS led to ROS-mediated autophagy. Periodontitis represents an example of how the organism responds to an insult. Here, some bacteria produce a local disease that may hasten the inflammatory systemic response, inducing and increasing autophagy [7]. In this situation, cell metabolism is triggered to counteract the aggression. The key organelle for energy production and autophagic control in the cell, the mitochondria, is activated [6]. In fact, it seems that mitochondrial 'health' should be fully considered when taking into account an organism's capacity to manage these pathological challenges. This may support the rising interest on the influence of mitochondria in inflammation-related diseases.

It is well known that the main source of cellular ROS is mitochondria. Moreover, it has been demonstrated that mitophagy/autophagy blockade leads to the accumulation of damaged ROS-generating mitochondria. This in turn activates the NLRP3 inflammasome which might explain the frequent association of mitochondrial damage with inflammatory diseases [29].

Recently, our group described that PBMCs from patients with periodontitis have a mitochondrial dysfunction characterized by lower CoQ_10 _levels and citrate synthase activity, together with high levels of ROS production [23]. Also, we described that LPS-treated gingival fibroblasts raised oxidative stress and led to mitochondrial dysfunction in terms of lower protein expression, loss of mitochondrial mass and impaired membrane potential. These results agree with data from the present study in which the influence of periodontitis in modifying systemic defense mechanisms, plus other local effects at the gingival level, leads to enhanced ROS production. It has been reported that ROS production and oxidative stress are a common consequence of dysfunctional mitochondria and play important roles in the development of autophagy [8]. We found increased expression of autophagy-related mRNA and proteins, demonstrating the activation of autophagy after ROS enhancement that occurred after mitochondrial dysfunction induced by *P. gingivalis *LPS. Moreover, lysosomal and autophagic markers (β-galactosidase, LC3 and LysoTracker staining) were higher in treated fibroblasts, indicating lysosomal proliferation. We confirmed these results by electron microscopy, which clearly showed the presence of laminar bodies and autophagosomes engulfing mitochondria.

Autophagy is a process by which cytosol and organelles are sequestered within double-membrane vesicles, delivering their contents for lysosome/vacuole degradation, followed by recycling of resulting materials [30]. The induction of autophagy could be part of the cellular program leading to cell death, or it could reflect attempts by the cell to repair itself through the removal of damaged organelles. In this sense, autophagy might be induced to aid in removing damaged mitochondria. In the present study, we observed an important activation of autophagy-related mRNA and proteins after *P. gingivalis *LPS induction. Furthermore, we also confirmed by immunofluorescence that autophagosome markers such as LC3, co-localized with cytochrome c, a mitochondrial marker, and β-galactosidase, a typical lysosomal enzyme. These results agree with previous studies in which LPS-induced inflammation led to autophagy overexpression, both in cultured cardiomyocytes of adult rats [31] as well as in rat liver tissue [32].

To test if LPS treatment activated autophagy via the induction of ROS production, we cultured LPS-treated HGF with CoQ_10_, α-tocopherol, BHA and NAC, all of them very efficient antioxidants. It is worthwhile to underline that CoQ_10 _could act as a key molecule in this context for cell well-being, both for its antioxidant properties [33] and for its essential redox role in the mitochondrial respiratory chain [34]. Results showed that all antioxidants significantly reduced acidic vacuoles induced by treatment with LPS. As stated before, we previously established the relationship between LPS treatment and HGF and ROS [23]. In a recent investigation, a similar finding was also described in hepatic mitochondria from mice treated with a single dosage of LPS. The authors found that LPS administration affected mtDNA and eventually mitochondrial function, while the use of antioxidant treatments with Mn-Superoxide Dismutase, nitric oxide synthase inhibitors, superoxide or peroxynitrite scavengers prevented the above mentioned effects. Noteworthy, in our study, is that treatment with antioxidants also significantly decreased conversion of LC3-I to LC3-II, suggesting a reduction in autophagosome formation. CoQ_10 _and α-tocopherol, both lipophilic antioxidants, were more efficient in significantly attenuating ROS production, thus confirming the importance of ROS generated in the lipophilic environment of mitochondrial membranes. In the work by Choumar *et al*. [34], a role of superoxide anion (O_2_•¯), reacting with nitric oxide to form mtDNA and protein-damaging peroxynitrite, was pointed out. Recently, O_2_•¯ has been proposed as the major ROS regulating autophagy [35]. These are new indications about the importance of proper preservation of structure and function of cell mitochondria. In this way, mitochondrial damage might lead to further enhanced ROS production, resulting in a downward spiral where mitochondrial viability is concerned. In turn, the accumulation of dysfunctional mitochondria is a very critical step because it is related to aging, cancer and neurodegenerative diseases [36].

Autophagy is like a double-edged sword, playing a role in cell survival as well as in cell death. It promotes cell death in some settings, but acts as a protective response in others. Thus, it is believed that selective mitochondrial autophagy (mitophagy) contributes to the maintenance of mitochondrial quality by eliminating damaged mitochondria or their excessive number [34], although little is known about this mechanism. It has been proposed that autophagy might act as an adaptive mechanism, defending organisms against the inflammatory process, and could be the background converging point with CVD. It may constitute an important physiological or pathophysiological response to cardiac stress, such as ischemia or pressure overload, which are frequently encountered in patients with coronary artery disease, hypertension, aortic valvular disease and congestive heart failure. The accumulation of autophagosomes has been noted in cardiac biopsy tissues of patients with these disorders, rodent models of these cardiac diseases, and isolated stressed cardiomyocytes [37]. Inhibition of autophagy in the heart induces age-related cardiomyopathy in experimental animals [38]. By contrast, induced autophagy in atherosclerosis plaque cells is a survival pathway in plaque stability and rupture [39]. Consistent with what has been mentioned above, previous studies have supported the hypothesis that autophagy has a protective role in LPS-induced injury in cardiomyocytes [40]. In agreement with this hypothesis on the protective role of autophagy, the present research demonstrates that disruption of autophagic processing by 3-MeA leads to cell death.

Given our results, we could hypothesize that mitochondrial dysfunction could represent a possible common functional derangement linking different inflammatory diseases such as periodontitis and CVD. In this sense, it could be a common event in all patients with periodontitis, namely a possible risk factor: in fact, mitochondria play an important role in proinflammatory signaling and ROS production that has also been shown to be an important activator of inflammasome-mediated inflammation [28]. Autophagic turnover of cellular constituents, either general or specific for mitochondria (that is, mitophagy), eliminates dysfunctional or damaged mitochondria, thus counteracting degeneration, dampening inflammation and preventing unwarranted cell loss. To the best of our knowledge, this is the first time autophagy activation has been described in patients with periodontitis.

## Conclusions

The demonstrated importance of autophagy in inflammatory conditions such as CVD, together with the role that this physiological process exerts in infective conditions, should be considered in relation to public health management. Control of autophagy has been considered as a new therapeutic approach in CVD and cancer [8]. Results from the present study suggest that autophagy is also an important mechanism in other conditions related to inflammation or alterations of the immune system, such as periodontitis. The link between periodontitis and CVD has been fully established in recent years. In fact, the mouth is a very accessible part of the body, offering an easy way of obtaining biological samples such as saliva or epithelial cells. Accordingly, HGFs could represent a good way to test the systemic status of the organism in relation to autophagy and consequently to understand more about inflammation and inflammatory related diseases.

## Abbreviations

3-MeA: 3-methyl adenine; ATGs: autophagy-related genes; BHA: butylated hydroxyanisole; CAL: clinical attachment level; CoQ_10_: coenzyme Q_10_; CVDs: cardiovascular diseases; FBS: fetal serum bovine; GM: recession of the gingival margin; HGF: human gingival fibroblasts; LPS: lipopolysaccharide; NAC: N-acetylcysteine; O_2_•¯: superoxide anion; PBS: phosphate-buffered saline; PCR: polymerase chain reaction; PD: periodontal probing depth; PMBCs: peripheral blood mononuclear cells; ROS: reactive oxygen species; RT: reverse transcriptase.

## Competing interests

The authors declare that they have no competing interests.

## Authors' contributions

Study concept and design: PB and MB. Acquisition of data: MDC, AGA, SA and RVM. Analysis and interpretation of data: PB, JLQ, MCRT, MdM and MB. Drafting of the manuscript: PB, MDC, JLQ and MB. Critical revision of the manuscript for important intellectual content: PB, JLQ and MB. Obtained funding: PB. Study supervision: PB and MB. All authors have read and approved the final manuscript.

## Pre-publication history

The pre-publication history for this paper can be accessed here:

http://www.biomedcentral.com/1741-7015/10/122/prepub
